# Of Course, Breaking Bad News Is Not Just for Patients: A Quality Improvement Project Survey to Explore How Staff Are Informed of Patient Deaths

**DOI:** 10.1192/bjo.2024.409

**Published:** 2024-08-01

**Authors:** Sian Davies, Kishan Pankhania, Imran Waheed

**Affiliations:** Birmingham and Solihull Mental Health NHS Foundation Trust, Birmingham, United Kingdom

## Abstract

**Aims:**

Mental health professionals are very likely to experience a patient death at least once in their careers. The Royal College of Psychiatrists published a framework for supporting mental health staff following the death of a patient by suicide. It states that ‘how the news about a patient's suicide is imparted influences the emotional impact of the death and is therefore very important'. We sought to explore how healthcare professionals are informed about patient deaths within Birmingham and Solihull Mental Health NHS Foundation Trust.

**Methods:**

A 25-question survey was devised to explore how staff have been informed about patient deaths and the impact it had on them. This was emailed to all grades of doctors, nurses and allied healthcare professionals within the trust and results were collected over 11 days.

**Results:**

83 healthcare professionals completed the survey.72 respondents had experienced a patient death within the trust. Of 72, 48.6% of respondents felt they had not been informed about their most recent patient death in a sensitive manner. There was wide variability in the method by which staff were first informed. 27.8% of respondents first learned of the patient's death via an email from the trust lawyers, patient safety team or another party. Of these 20, 17 felt they had not been informed in a sensitive manner. 63.9% (n = 46) reported that they had not been signposted to any support. Qualitative data suggested that the way in which people were informed had a wide-ranging impact. Many respondents felt shocked and upset. With hindsight, people would have appreciated being informed face to face and being given time to reflect. Of the total 83 responses, 82% (n = 68) felt that there should be a specific policy about how staff are informed about patient deaths.

**Conclusion:**

Results from this survey demonstrate a large scope for improvement in the way that staff are informed of patient deaths. Using these results, we will generate change ideas for a quality improvement project which aims to inform staff more sensitively about patient deaths. Feedback suggested implementation of a new protocol to guide team managers and consultants on how to inform staff; it may help to standardise the process and the support provided. Patient deaths have a significant impact on the mental health of staff; communicating this matter compassionately may help to alleviate immediate feelings of distress and mediate the long-term impact on staff wellbeing and satisfaction.

